# Prognostic assessment of hypoxia and metabolic markers in cervical cancer using automated digital image analysis of immunohistochemistry

**DOI:** 10.1186/1479-5876-11-185

**Published:** 2013-08-08

**Authors:** Bo Wook Kim, Hanbyoul Cho, Joon-Yong Chung, Catherine Conway, Kris Ylaya, Jae-Hoon Kim, Stephen M Hewitt

**Affiliations:** 1 Tissue Array Research Program & Applied Molecular Pathology Lab, Laboratory of Pathology, Center for Cancer Research, National Cancer Institute, National Institutes of Health, Bethesda, MD 20892, USA; 2 Department of Obstetrics and Gynecology, Gangnam Severance Hospital, Yonsei University College of Medicine, 146-92 Dogok-Dong, Gangnam-Gu, Seoul 135-720, Korea; 3 Department of Obstetrics and Gynecology, Korea Cancer Center Hospital, Seoul 139-706, Korea

**Keywords:** HIF-1α, c-Met, Cervical cancer, Prognosis, Automated digital image analysis

## Abstract

**Background:**

Hypoxia inducible factor-1 alpha (HIF-1α), induced by tumor hypoxia, regulates tumor cell metabolism and metastasis by up-regulation of c-Met, carbonic anhydrase 9 (CA9) and glucose transporter 1 (GLUT1). The prognostic significance of hypoxia and metabolic markers is not clearly defined in cervical cancer. Here, we have examined the primary players in the hypoxia signaling pathway, by immunohistochemistry, but confirming their interactions, as well as defining which proteins are associated with outcome.

**Methods:**

The study subjects were comprised of cervical intraepithelial neoplasia (CIN, *n *= 209), carcinoma *in situ* (CIS, *n *= 74), cervical cancer (*n *= 179), and matched nonadjacent normal tissues (*n *= 357). Immunohistochemistry (IHC) was performed to identify HIF-1α, c-Met, CA9, and GLUT1. IHC scoring was performed using automated digital image analysis and the association of hypoxic markers with prognostic outcome was evaluated.

**Results:**

HIF-1α, c-Met, CA9 and GLUT1 expression were higher in cervical cancer than in CIN and normal cervix (all *P* < 0.001). Among these markers, expression of HIF-1α and c-Met were significantly different in FIGO stage (*P* < 0.001 and *P* = 0.019, respectively) and patients with lymph node metastasis (*P* < 0.001 and *P* = 0.010, respectively). HIF-1α expression was correlated with c-Met expression in cervical cancer (*P* < 0.001). High expression of HIF-1α and c-Met showed worse 5-year overall survival rate (*P* = 0.047 and *P* = 0.005, respectively) than low expression group, but CA9 and GLUT1 did not show significant survival difference. After adjusting the prognostic covariates, c-Met was found to be an independent risk factor (HR=3.27; 95% CI, 1.05-10.23, *P* = 0.041) for overall survival in cervical cancer.

**Conclusions:**

We demonstrate that c-Met correlates with HIF-1α and is a poor prognostic factor in survival in cervical cancer.

## Background

Cervical cancer is the third most common cancer in women worldwide and remains a significant cause of morbidity and mortality in developing countries including South America, sub-Saharan Africa, and the South-Central Asia [1]. Many developed countries have achieved significant successes in reducing the burden of cervical cancer through substantial health care investments for screening programs and diagnostic workup. On the other hand, cervical cancer is the leading cancer among women in many resource-constrained settings of developing countries, where incidence and mortality rate are about five- to six-times higher [1]. Overall the clinical management of patients with premalignant and malignant cervical disease is a significant burden on the health care system. Although tremendous progress has been made in the diagnosis and treatment of cervical cancer, there is still a need for clinically robust biomarkers to further refine the screening, triage and management of women.

Hypoxia is present in a wide range of solid tumors and is often associated with tumor glycolysis, angiogenesis, and poor prognosis, as well as invasion and metastasis by activating relevant gene expression through hypoxia inducible factor-1α (HIF-1α) [2-6]. In normoxic condition, HIF-1α is degraded within minutes through the interaction of iron and oxygen-dependent von Hippel-Lindau tumor suppressor (VHL) [7]. However, in hypoxic condition or in the absence of VHL, HIF-1α is stabilized and binds to DNA on the hypoxia response elements contained within the promoter regions of various target genes [3,8]. HIF-1α synthesis is increased by various growth factors, cytokines and other signaling molecules responsible for stimulating phosphatidylinositol 3-kinase (PI3K) or mitogen-activated protein kinase (MAPK) pathways [8]. Increased HIF-1α activates target genes involved in tumor cell growth, angiogenesis, metabolism, apoptosis and metastasis [4,9]. In a hypoxic cancer microenvironment, a metabolic shift occurs from an oxidative to glycolytic metabolism. HIF-1α converts pyruvate metabolism in mitochondria to cytoplasmic alteration of pyruvate into lactic acid [10]. In cytoplasmic glucose metabolism, high glucose consumption takes place in tumor cells and glucose transporter type 1 (GLUT1) is involved in delivering glucose into these tumor cells. Cytoplasmic glucose metabolism produces lactic acid that induces acidosis, but carbonic anhydrase 9 (CA9) regulates cell pH through Cl-/HCO3- exchanger uptake of HCO3-. c-Met, known as hepatocyte growth factor receptor, is activated by HIF-1α and it is known for tumor invasion and metastasis [5,6].

HIF-1α and its regulated markers including c-Met, CA9 and GLUT1 were reported to be highly expressed in various cancers and associated with poor prognosis [3]. However, the relationship of these hypoxia markers in the prognosis of cervical cancer is unclear [11,12]. Furthermore, hypoxia results in resistance against radiation and chemotherapy which are treatment options in cervical cancer, so prognostic significance of hypoxia markers must be clarified. Immunohistochemistry (IHC) utilizes antigen-antibody recognition in detecting specific antigens within tissues and it's widely used in surgical pathology. A variety of markers has been evaluated for their role in the diagnosis, prognosis, and prediction of treatment. However, interpretation of immunohistochemical stains by traditional-microscope based review introduces inter and intra observer variability and remains a challengeable issue [13]. In this context, several automated image analysis systems have been developed and their use has shown promising advantages including objectivity, speed, reproducibility, and accurate quantitative assessment of immunohistochemical stains [14,15]. In the current study, the expression of HIF-1α and functionally related proteins, including c-Met, CA9 and GLUT1, were analyzed in cervical cancer patients by combined IHC and automated digital image analysis. Furthermore, we evaluated the association of these markers with prognosis.

## Materials and methods

### Patient selection

One hundred seventy nine paraffin-embedded specimens of cervical cancer, 74 carcinoma *in situ* (CIS), 209 cervical intraepithelial neoplasia (CIN), and 357 matched normal tissues obtained from Gangnam Severance Hospital, Yonsei University College of Medicine in Seoul, Korea and the Korea Gynecologic Cancer Bank through Bio & Medical Technology Development Program of the Ministry of Education, Science and Technology, Korea between 1996 and 2010 were included in the study. Medical records were reviewed to collect patient data including age, cancer stage, tumor differentiation, cell type, tumor size, lymphovascular space invasion (LVSI) and lymph node (LN) metastasis. The tumors were staged according to the FIGO stage and histologically classified and graded according to WHO grade. Patients with operable condition underwent radical hysterectomy with pelvic and aortic lymph node dissection, and concurrent chemoradiation therapy was added to the patient with risk factors such as LN metastasis, parametrial invasion and positive resection margin. The patients with inoperable condition underwent radiation or chemoradiation therapy. The current study was approved by the Institutional Review Board of Gangnam Severance Hospital.

### Tissue microarray construction and immunohistochemistry

Tissue microarrays (TMAs) were constructed using a tissue arrayer (Pathology Devices, Westminster, MD). Briefly, slides were reviewed by a pathologist and areas containing each category were annotated on the H&E slides. Four cylindrical tissue cores of 1.0 mm diameter, consisting of matched tumor specimen and normal epithelial tissue, were then taken from the corresponding regions of the paraffin blocks and transplanted into a recipient paraffin block.

For immunohistochemical staining, all paraffin-embedded sections were cut at 5 micron thickness, deparaffinized through xylene and dehydrated with graded ethanols. Antigen retrieval was performed in heat-activated antigen retrieval pH 6 (Dako, Carpinteria, CA) for HIF-1α and antigen retrieval pH 9 (Dako) for c-Met, CA9, and GLUT1, respectively, then specimens were incubated with 3% H_2_O_2_ for 15 min. Non-specific binding blocked with protein block (Dako) for 20 min at room temperature. The sections were incubated with anti- HIF-1α antibodies (Novus Biologicals, Littleton, CO, Clone ESEE122) at 1:2000 for 30 minutes, anti-GLUT1 antibodies (NeoMarkers, Fremont, CA, Clone SPM498) at 1:3000 overnight, anti-CA9 antibodies (M75 antibody kindly provided by Dr S. Pastorekova) at 1:100 for 2 hours and anti-c-Met antibodies (Abcam, Cambridge, MA, Clone EP1454Y) at 1:500 for 30 minutes, respectively. Subsequently, sections were incubated with DAKO Env^+^ secondary antibody for 30 min, visualized with 3,3-diaminobenzadine for 10 minutes for chromogenic development, washed and counterstained with hematoxylin. Appropriate negative controls were concurrently performed, and the TMAs included appropriate positive control tissues.

### Digital image analysis

Immunohistochemically stained sections were digitized at 20 × magnification utilizing an Aperio Scanscope CS (Aperio, Vista, CA). The Aperio Scanscope CS obtains 20 × images with a spatial resolution of 0.45 μm/pixels. Images were reviewed utilizing an online software application, Digital Image Hub (SlidePath, Dublin, Ireland). Digital Image Hub enabled users to annotate normal and tumor regions. Once the areas were annotated, they were sent for automated image analysis utilizing TissueIA (SlidePath’s Tissue IA system, version 3.0). Within Tissue IA, an algorithm was developed to quantify cytoplasmic HIF-1α and membranous c-Met, CA9 and GLUT1. HIF-1α was mainly stained in the cytoplasm, but partial nucleus staining was included in the analysis. The output from the algorithm returns a number of quantitative measurements, namely the intensity, concentration and percentage of positive staining present. Quantitative scales of intensity and percentage were categorized to 4 and 5 classes, respectively, after cut-off values were determined. The intensity of staining was categorized as 0 (no staining), 1+ (weak), 2+ (moderate) and 3+ (strong), and the percentage of staining was categorized as 0 (≤ 5%), 1+ (6-25%), 2+ (26-50%), 3+ (51-75%) and 4+ (> 75%). Final IHC score was calculated from a combination of intensity and percentage score (Range 0–12) [16].

### Statistical analysis

IHC scores were compared using one-way ANOVA test and independent t-test. Chi-square test and Spearman's rank correlation analysis were used to evaluate the association between hypoxia related markers. Immunohistochemical cut-off for high expression of tumor markers was determined through the receiver operating characteristic (ROC) curve analysis. The sensitivity and specificity for discriminating death or alive was plotted at each IHC score, thus generating a ROC curve. The cut-off value was established to be the point on the ROC curve where the sum of sensitivity and specificity was maximized [17]. Kaplan-Meier survival analysis was performed to determine the association of HIF-1α or c-Met expression with disease-free and overall survival, and the survival curves were compared between groups using log-rank tests. Univariate and multivariate analyses of hazard ratio for death were performed using Cox proportional hazards regression. Statistical analyses were performed using SPSS version 19.0 (SPSS Inc., Chicago, IL). A value of *P* < 0.05 was considered statistically significant.

## Results

### Clinicopathological characteristics of patient cohort

Table 1 summarizes patient’s clinicopathological characteristics. In 179 patients with cervical cancer, 123 patients of stage I, 51 of stage II and 5 of stage IV were included. The ages of the patients ranged from 19 to 83 years (mean, 43.8 years). The tumor sizes ranged from 0.3 to 12 cm (mean, 2.7 cm). The cervical cancers comprised 144 squamous cell carcinomas (SCC), 19 adenocarcinomas, 8 adenosquamous and 8 other types (4 small cell carcinomas, 2 neuroendocrine and 2 mixed cell types). At the time of last follow-up, recurrence had occurred in 32 of 179 (17.8%) of the cohort. 179 patients were evaluated for survival analysis and the mean follow-up time of surviving patients was 55.6 months (range 6 and 60), 17 patients (10%) died during the follow-up period.

**Table 1:** Characteristics of patients

**Variable**	**Frequency**	**%**
**Age**	43.8^a^	
**Diagnostic category**		
Low grade CIN	65	14.1
High grade CIN	144	31.2
CIS	74	16.0
Cervical cancer	179	38.7
**FIGO stage**		
I	123	68.7
II	51	28.5
IV	5	2.8
**Tumor differentiation**^b^		
Well	2	1.2
Moderate	125	73.1
Poor	44	25.7
**Cell type**		
Squamous cell carcinoma	144	80.4
Adenocarcinoma	19	10.6
Adenosquamous	8	4.5
Others	8	4.5
**Tumor size**		
< 4 cm	130	72.6
≥ 4 cm	49	27.4
**Lymphovascular invasion**^**c**^		
Negative	93	57.1
Positive	70	42.9
**Lymph node metastasis**^d^		
Negative	124	75.2
Positive	41	24.8

CIN, cervical intraepithelial neoplasia; CIS, carcinoma *in situ*; FIGO, International Federation of Gynecology and Obstetrics; ^a^mean value, ^b^calculated only 171 cases with available information of growth pattern, ^c^calculated only 163 cases with available information of examined lymphovascular invasion, ^d^calculated only 165 cases with available information of examined lymph node.

### Expression of hypoxic and metabolic markers

**Figure 1:**
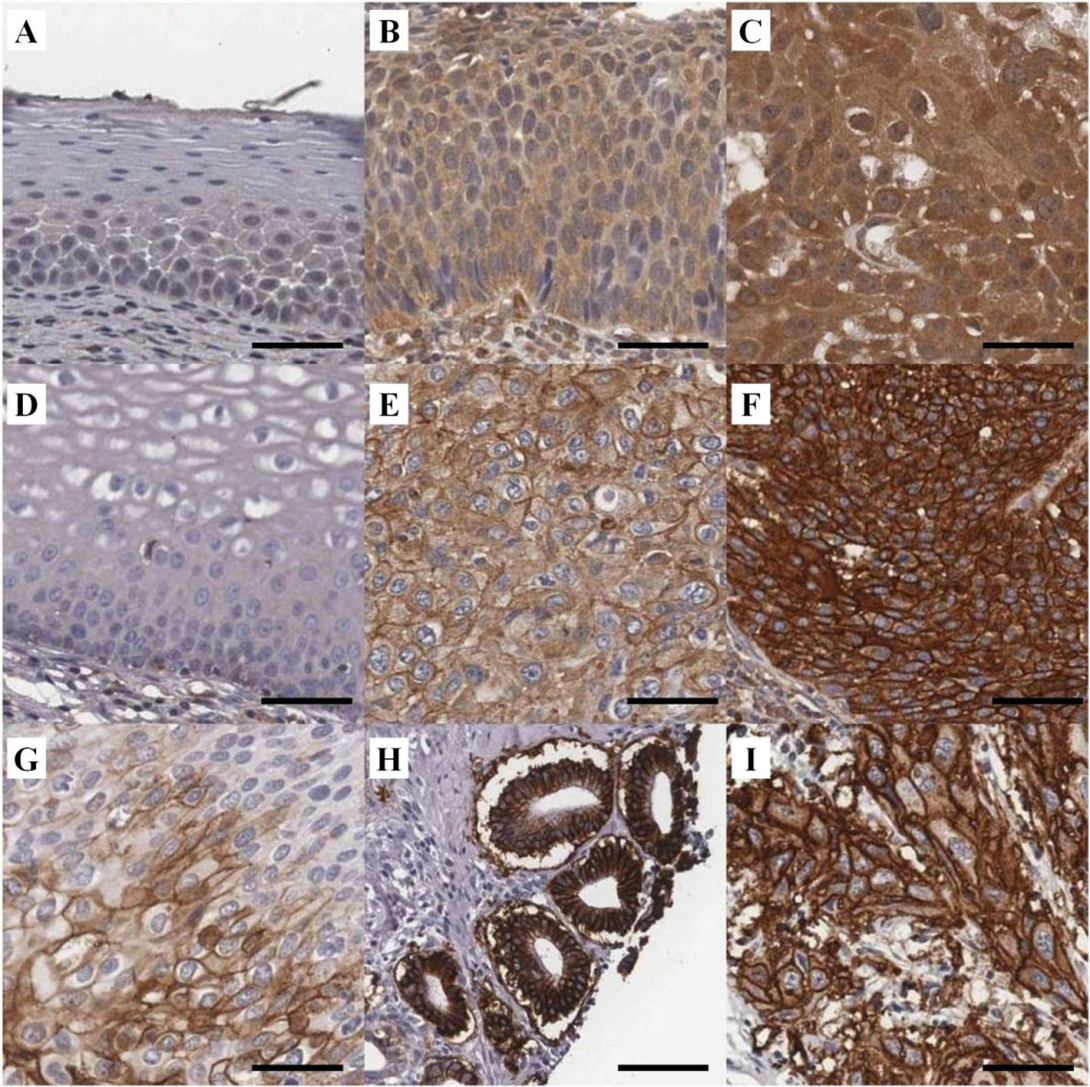
**Representative immunohistochemical expression for HIF-1α, c-Met, CA9 and GLUT1.** HIF-1α is stained in cytoplasm shown with no staining in normal cervix **(A)**, weak staining intensity in high grade CIN **(B)**, and strong staining intensity in squamous cell carcinoma **(C)**. c-Met **(D**-**F)**, CA9 **(G**, **H)** and GLUT1 **(I)** shows cell membranous staining. Representative c-Met expression in cervical samples shown with no staining in normal cervix **(D)**, weak membranous staining intensity in squamous cell carcinoma **(E)** and strong intensity in squamous cell carcinoma **(F)**. CA9 expression showing moderate intensity staining in carcinoma *in situ* (CIS) **(G)** and strong staining in adenocarcinoma **(H)**. GLUT1 expression showing strong intensity in squamous cell carcinoma **(I)**. Scale bar: 50 μm.

**Figure 2:**
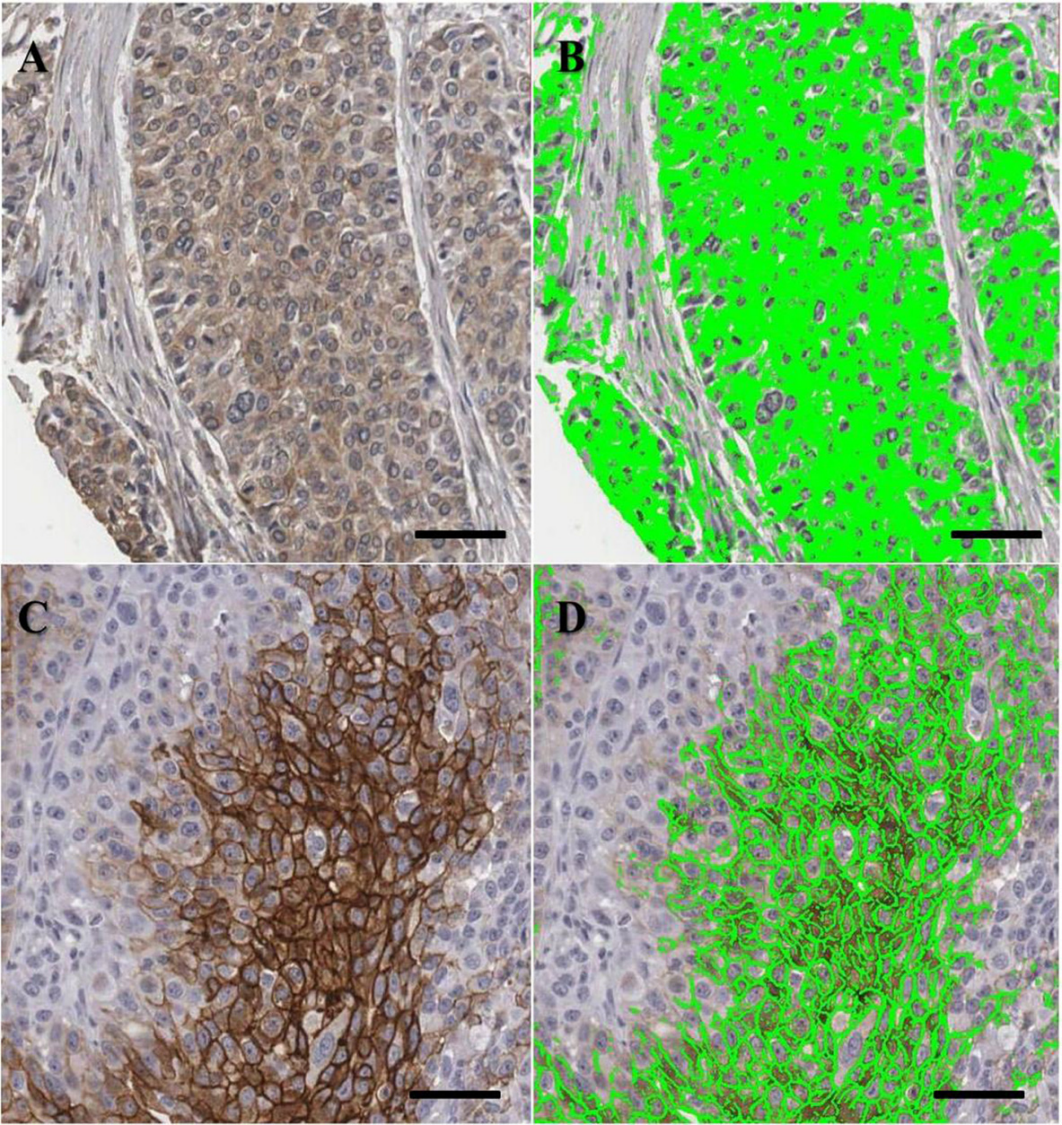
**Digital image analysis of cytoplasmic and membranous staining.** Cytoplasmic HIF-1α staining is shown **(A)** and automated image analysis utilizing TissueIA recognizes cytoplasmic HIF-1α staining highlighted in green color **(B)**. CA9 is shown in membranous staining **(C)** and automated image analysis determines membranous CA9 staining highlighted in green color **(D)**. The output from the algorithm returns a number of quantitative measurements for intensity and percentage of positive staining present. Scale bar: 100 μm.

We examined expression of HIF-1α, c-Met, CA9, and GLUT1 in cervical neoplasias and cancer specimens by IHC. Subsequently, we performed analysis of these markers using automated digital image software. Representative immunohistochemical expression of HIF-1α, c-Met, CA9 and GLUT1 are presented in Figure 1. As shown Figure 1, c-Met (Figure 1D-1F), CA9 (Figure 1G and 1H) and GLUT1 (Figure 1I) expression was observed in the tumor cell membrane, while HIF-1α expression (Figure 1A-1C) was mainly observed in the cytoplasm, with some cases also demonstrating weak nucleus staining. The examples of IHC and digital image analysis output images are presented in Figure 2. The green color represents what is classified as positively stained by the algorithms.

The TMA contains 179 cases of cervical cancer, however due to the complexity of sectioning, staining, as well as heterogeneity of the samples, between 144 and 162 of samples could be interpreted for the individual markers. One hundred fifty one (84.4%, HIF-1α), 152 (84.9%, c-Met), 144 (80.5%, CA9), and 162 (90.5%, GLUT1) out of 179 cases were found suitable for IHC evaluation and their detailed IHC scoring pattern is shown in Table 2. A ROC analysis was plotted to investigate the optimal cut-off values that maximized the sum of sensitivity and specificity (Table 3). HIF-1α and c-Met showed statistically significant AUCs with 0.677 and 0.650, respectively (*P* = 0.015 and *P* = 0.040, respectively). However, CA9 and GLUT1 did not show significantly predictable point for death (*P* = 0.993 and *P* = 0.322, respectively). 60 of 151 cancers (39.7%) had high expression (cut-off value: 8) of HIF-1α, 42 of 152 cancers (27.6%) had high expression (cut-off value: 8) of c-Met, 45 of 144 cancers (31.3%) had increased expression (cut-off value: 2) of CA9, 37 of 162 (22.8%) had elevated expression (cut-off value: 8) of GLUT1. Clinicopathological characteristics of HIF-1α, c-Met, CA9 and GLUT1 expression are summarized in Table 2. HIF-1α, c-Met, CA9 and GLUT1 expression correlated significantly to diagnostic category (Table 2). HIF-1α expression were also correlated significantly to FIGO stage (*P* < 0.001), tumor cell type (*P* = 0.010), LN metastasis (*P* < 0.001). However, there was no statistically significant difference in HIF-1α expression with regard to tumor differentiation, tumor size and LVSI (Table 2). Statistically significant correlation was found between c-Met expression and FIGO stage (*P* = 0.019), LVSI (*P* = 0.001) and LN metastasis (*P* = 0.010), while there was no association between c-Met expression and tumor differentiation, tumor cell type and tumor size (Table 2). CA9 immunoreactivity was elevated in non-squamous cell type (*P* = 0.040) whereas GLUT1 expression was significantly increased in squamous cell type (*P* = 0.001). GLUT1 was highly expressed in negative LN metastasis compared to positive LN metastasis (*P* = 0.001).

**Table 2:** Association of HIF-1α, c-Met, CA9 and GLUT1 IHC expression with clinicopathological characteristics in cervical cancer

	**HIF-1α**			**c-Met**			**CA9**			**GLUT1**		
	**N**	**Mean score (95% CI)**	***p value***	**N**	**Mean score (95% CI)**	***p value***	**N**	**Mean score (95% CI)**	***p value***	**N**	**Mean score (95% CI)**	***p value***
**Diagnostic category**			*< 0.001*			*< 0.001*			*< 0.001*			*< 0.001*
Normal ^a^	313	4.4 (4.0-4.7)		346	0.4 (0.3-0.6)		314	0.0 (0.0-0.0)		357	0.9 (0.7-1.1)	
Low grade CIN ^b^	54	5.1 (4.3-5.9)		62	0.6 (0.2-1.0)		55	0.4 (0.0-0.8)		40	1.8 (0.9-2.7)	
High grade CIN ^b^	104	5.4 (4.9-6.0)		115	0.7 (0.4-0.9)		132	1.9 (1.3-2.5)		121	2.2 (1.8-2.7)	
CIS ^b^	56	5.7 (4.9-6.4)		60	1.7 (1.0-2.4)		69	1.3 (0.6-1.9)		73	2.4 (1.7-3.6)	
Cervical cancer ^b^	151	5.4 (4.9-6.0)		152	4.7 (4.0-5.3)		144	2.1 (1.5-2.8)		162	4.0 (3.3-4.6)	
**FIGO stage**			*< 0.001*			*0.019*			*0.149*			*0.243*
I	96	4.6 (4.0-5.2)		98	4.2 (3.4-5.0)		95	1.7 (0.9-2.4)		114	4.3 (3.5-5.1)	
II	50	6.9 (5.9-7.9)		49	5.2 (3.9-6.4)		45	2.9 (1.6-4.1)		43	3.1 (2.1-4.0)	
IV	5	6.0 (2.4-9.5)		5	9.2 (3.7-14.6)		4	4.0 (−5.0-13.0)		5	4.0 (0.4-7.5)	
**Tumor differentiation**			*0.350*			*0.195*			*0.298*			*0.169*
Well+Moderate	105	5.6 (4.9-6.2)		107	4.4 (3.7-5.2)		105	2.4 (1.6-3.1)		115	4.1 (3.3-4.8)	
Poor	40	5.0 (3.9-6.1)		40	5.4 (4.0-6.9)		37	1.5 (0.3-2.6)		41	3.1 (1.8-4.3)	
**Cell Type**			*0.010*			*0.325*			*0.040*			*0.001*
SCC	119	5.8 (5.2-6.4)		118	4.5 (3.7-5.2)		111	1.6 (1.0-2.3)		130	4.4 (3.7-5.2)	
Other	32	4.1 (2.8-5.4)		33	5.3 (3.7-6.9)		33	3.7 (1.8-5.5)		31	1.9 (0.9-2.8)	
**Tumor size**			*0.210*			*0.195*			*0.851*			*0.947*
< 4cm	104	5.2 (4.6-5.8)		106	4.9 (4.1-5.7)		100	2.2 (1.4-2.9)		119	4.0 (3.2-4.8)	
≥ 4cm	47	5.9 (4.9-7.0)		46	4.1 (2.9-5.3)		44	2.0 (0.9-3.1)		43	3.9 (2.8-5.0)	
**LVSI**			*0.725*			*0.001*			*0.998*			*0.801*
Negative	73	5.0 (4.2-5.7)		74	3.6 (2.7-4.4)		65	2.0 (1.0-3.0)		84	4.0 (3.1-4.9)	
Positive	62	5.1 (4.3-6.0)		62	5.9 (4.8-7.0)		63	2.0 (1.0-3.0)		63	4.3 (3.2-5.3)	
**LN metastasis**			*< 0.001*			*0.010*			*0.820*			*0.001*
Negative	100	4.4 (3.9-5.0)		102	4.0 (3.3-4.8)		95	2.0 (1.2-2.8)		113	4.5 (3.7-5.4)	
Positive	37	6.7 (5.5-7.9)		36	6.3 (4.8-7.9)		35	2.2 (0.8-3.6)		36	2.6 (1.6-3.6)	

Some data of tumor differentiation, LVSI and LN metastasis are not available on retrospective chart review. SCC, squamous cell carcinoma; LVSI, lymphovascular space invasion; ^a^ normal sample was constructed with nonadjacent normal epithelium from tumor in same patient, ^b^IHC staining of each marker was evaluated with available tumor cores from 209 CIN, 74 CIS and 179 cancer cores.

**Table 3:** Optimal cut-off value for high expression was determined by identifying point to predict death

			**Prediction of death**		
**Marker**	**AUROC (95% CI)**	***P value***	**Cut-off score**	**Sensitivity (%)**	**Specificity (%)**
**HIF-1α**	0.677 (0.534-0.819)	*0.015*	8	66.7	63.9
**c-Met**	0.650 (0.499-0.800)	*0.040*	8	61.1	76.9
**CA9**	0.501 (0.355-0.646)	*0.993*	2	23.5	82.7
**GLUT1**	0.424 (0.295-0.554)	*0.322*	8	68.7	34.9

### Association of hypoxic and metabolic markers

To determine the association between expression of HIF-1α, c-Met, CA9 and GLUT1, HIF-1α score values were compared with c-Met, CA9 and GLUT1 values using Chi-square test (data not shown) and Spearman’s rank correlation analysis (Table 4). Pre-invasive cervical lesions were evaluated for co-expression between HIF-1α and its regulated markers. In CIN and CIS specimens, HIF-1α expression showed significant correlation with GLUT1 (Chi-square test; *P* = 0.001, Spearman’s rho = 0.217, *P* = 0.005) whereas HIF-1α expression was not correlated with those of CA9 (Chi-square test; *P* = 0.043, Spearman’s rho = 0.097, *P* = 0.195) and c-Met (Chi-square test; *P* = 0.136, Spearman’s rho = 0.137, *P* = 0.062) (data not shown). Of 179 cervical cancer specimens, 148 (82.6%) was available to confirm co-expression between HIF-1α and c-Met, 131 (73.1%) was available between HIF-1α and CA9, and 137 (76.5%) was available between HIF-1α and GLUT1 (Table 4). In CIN and CIS of 283 samples, co-expression of HIF-1α was evaluated with c-Met (*n *= 186, 65.7%), CA9 (*n *= 181, 64.0%) and GLUT1 (*n *= 167, 59.0%), respectively. Expression of HIF-1α was significantly correlated with that of c-Met (*P* < 0.001 and Spearman’s rho = 0.351) in cervical cancer specimens whereas HIF-1α expression showed no association with CA9 and GLUT1 expression in cervical cancer.

**Table 4:** Correlation of HIF-1α, c-Met, CA9 and GLUT1 IHC expression in cervical cancer

	**HIF-1α**			**c-Met**			**CA9**			**GLUT1**		
	***n***	***r***	***P***	***n***	***r***	***P***	***n***	***r***	***P***	***n***	***r***	***P***
**HIF-1α**	-	-	-	148	0.351	*<0.001*	131	0.147	0.094	137	0.034	0.690
**c-Met**	148	0.351	*<0.001*	-	-	-	134	0.135	0.119	139	0.048	0.575
**CA9**	131	0.147	0.094	134	0.135	0.119	-	-	-	132	−0.108	0.216
**GLUT1**	137	0.034	0.690	139	0.048	0.575	132	−0.108	0.216	-	-	-

*r,* correlation coefficient; *P*, *p* value.

### Survival outcome of hypoxia and metabolic markers

**Figure 3:**
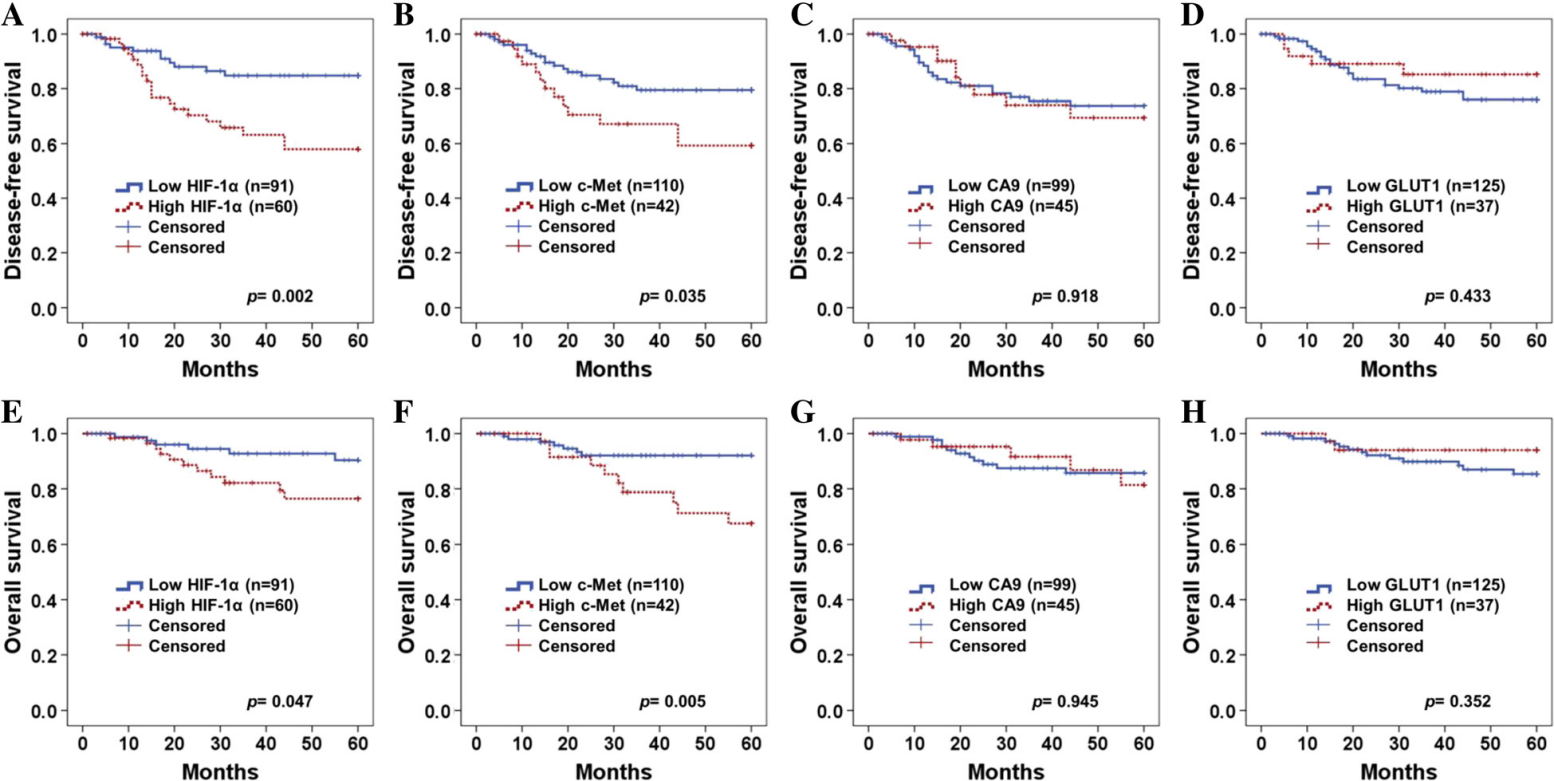
**Kaplan-Meier plots of disease-free survival (A - D) and overall survival (E – H) for categorized by HIF-1α (A &E), c-Met (B &F), CA9 (C &G) and GLUT1 (D &H).** High HIF-1α expression shows worse disease-free survival **(A**, *P* = 0.002**) **and overall survival rate **(E**, *P* = 0.047**)** than low expression (*P* = 0.047). Patients with high c-Met expression displayed shorter disease-free (*P* = 0.035) and overall survival (*P* = 0.005) compared with that of patients with low c-Met expression **(B** &**F)**. *P* values for two-sided log-rank statistics are given for each plot. Cut-off value of HIF-1α, c-Met, CA9 and GLUT1 are 8, 8, 2 and 8 of IHC score, respectively.

**Figure 4:**
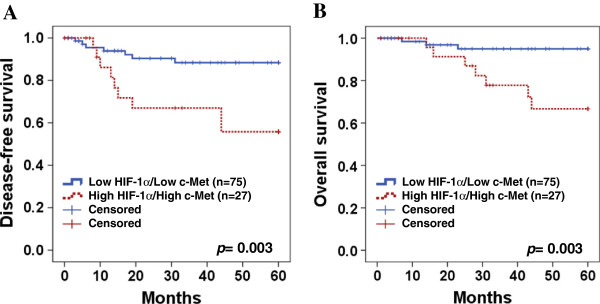
**Kaplan-Meier analysis of a combination of HIF-1α and c-Met expression.** High HIF-1α/high c-Met revealed a significantly difference on disease-free survival **(A, *P *= 0.003)** and overall survival **(B, *P *= 0.003)** compared to low HIF-1α/low c-Met. *P* values for two-sided log-rank statistics are given for each plot.

Five year disease-free survival and overall survival were analyzed through the Kaplan-Meier plots as shown in Figure 3. In survival analysis of HIF-1α, there were 20 recurrences and 11 deaths in 60 high expression patients, while 11 recurrences and 6 deaths in 91 low expression patients were shown during 5 year follow-up period. Mean 5-year overall survival time was 56.9 and 51.9 months in low HIF-1α expressions and high HIF-1α expressions, respectively. High expression of HIF-1α showed worse disease-free survival and overall survival rate than those of low expression group (*P* = 0.002 and *P* = 0.047, respectively) (Figure 3A & E). In survival analysis with c-Met expression, 13 recurrences and 10 deaths in 42 c-Met high expressions occurred, while 18 recurrences and 7 deaths were observed in 110 low expressions. Mean 5-year overall survival time was 56.5 months in low c-Met expression and 50.8 months in high c-Met expression. High expression of c-Met showed poorer disease-free and overall survival rate than low expression group with significant difference (*P* = 0.035 and *P* = 0.005, respectively) (Figure 3B & F). However, CA9 and GLUT1 did not show significant difference of disease-free survival (Figure 3C and D) and overall survival (Figure 4G & H) between high and low expression. When survival of patients with expression of high HIF-1α/high c-Met was compared with that of those with low HIF-1α/low c-Met, Kaplan-Meier analysis revealed a significant difference on both disease-free survival (*P* = 0.003, Figure 4A) and overall survival (*P* = 0.003, Figure 4B).

### Univariate and multivariate analysis

Cox proportional hazards analysis was performed to compare the impact of hypoxia and metabolic markers on survival with those of currently used clinicopathological prognostic factors (FIGO stage, tumor differentiation, tumor size, LVSI, LN metastasis) (Table 5). FIGO stage (*P* = 0.043), LN metastasis (*P* = 0.008), HIF-1α (*P* = 0.019) and c-Met (*P* = 0.005) showed significant hazard ratio in univariate analysis. We performed multivariate analysis with separate HIF-1α and c-Met, because both factors are dependent on each other. In multivariate analysis, LN metastasis (*P* = 0.08 and *P* = 0.038 for HIF-1α and c-Met, respectively) and c-Met showed statistical significance in overall survival (*P* = 0.041). Meanwhile, HIF-1α did not show significance under the multivariate analysis.

**Table 5:** Cox proportional univariate and multivariate analyses of the association between prognostic variables and overall survival in cervical cancer

	**Univariate analysis**		**Multivariate analysis based on HIF-1α**^a^		**Multivariate analysis based on c-Met**^a^	
	**Hazard ratio [95% CI]**	***p value***	**Hazard ratio [95% CI]**	***p value***	**Hazard ratio[95% CI]**	***p value***
**FIGO stage (≥IIb)**	2.60 [1.02-6.56]	*0.043*	1.03 [0.31-3.40]	*0.951*	0.96 [0.28-3.25]	*0.951*
**Tumor grade (poor)**	1.39 [0.55-3.51]	*0.486*	NA		NA	
**Tumor size (> 4 cm)**	2.33 [0.91-5.94]	*0.076*	NA		NA	
**LVSI**	2.81 [0.99-7.94]	*0.050*	0.91 [0.19-4.35]	*0.915*	0.79 [0.17-3.59]	*0.768*
**LN metastasis**	4.96 [1.75-13.99]	*0.008*	4.23 [0.84-21.34]	*0.080*	4.83 [1.09-21.42]	*0.038*
**HIF-1α**^b^	2.91 [1.09-7.78]	*0.019*	1.84 [0.54-6.30]	*0.326*	NA	
**c-Met**^c^	1.96 [1.22-3.16]	*0.005*	NA		3.27 [1.05-10.23]	*0.041*

NA, not applicable; LVSI, lymphovascular space invasion; ^a^ Separate multivariate analysis for HIF-1α and c-MET was performed because both factors are dependent on each other, ^b^ Cut-off value of HIF-1α is 8 of IHC score, ^c^ Cut-off value of c-Met is 8 of IHC score.

## Discussion

Expression of HIF-1α, c-Met, CA9 and GLUT1 were investigated in the current study. In those markers, HIF-1α and c-Met were associated with LN metastasis and FIGO stage in cervical cancer. HIF-1α and c-Met have been known as contributing factors for cancer invasion and LN metastasis in various cancers [18-20]. Activated HIF-1α under hypoxia reduces tissue integrity through the loss of E-cadherin, a cell adhesion molecule that acts as a suppressor of invasion and metastasis [21]. Conversely, inhibition of HIF-1α with siRNA induces E-cadherin expression, which increases cell to cell adhesion [21]. HIF-1α also facilitates disruption of basement membrane and extracellular matrix which are physical barriers against tumor cell migration [11]. After HIF-1α disrupts cell integrity and membrane, c-Met enhances tumor cell to invade surrounding stroma and migrate into the blood and lymphatic vessels [5,22].

We investigated correlation of HIF-1α expression with c-Met, GLUT1, or CA9 expression, respectively. Firstly, HIF-1α expression was associated with c-Met expression in cervical cancer, while no correlation between HIF-1α and c-Met was observed in CIN and CIS. Although c-Met is well known to be regulated by HIF-1α in *in vitro* study, its association has not been confirmed with cervical cancer. In our knowledge, this is the first report of correlation between HIF-1α and c-Met in cervical cancer tissue. For correlation between HIF-1α and c-Met in other cancer tissues, only limited number of pancreatic tissue showed tendency of correlation with c-Met and HIF-1α expression [23]. Furthermore, correlation between HIF-1α and c-Met is not reported in pre-invasive lesions as well. As for pre-invasive cervical lesions, not only was c-Met found to lack correlation with HIF-1α but also expressed much lower. Since CIN primarily comprises of cells that proliferate without the feature of metastasis and invasion, c-Met expression in these cells is expected to remain low. Regulating factors such as TGF-β, proteasomal inhibitor, AP1, PI3K and ERK are involved in the CA9 expression, which are independent of HIF-1α [24-26].

In this study, c-Met was an independent prognostic factor on overall survival in cervical cancer. Similar to our study, c-Met is associated with FIGO stage and LN metastasis, and c-Met expression influences on the adverse survival outcome in cervical cancer [20,27,28]. Although the high expression of HIF-1α revealed worse 5-year overall survival on Kaplan-Meier plots, HIF-1α did not show significance on multivariate analysis. In previous studies performed on patients with cervical cancer, prognostic significance of HIF-1α was inconsistent, even though the HIF-1α is involved in tumor cell metabolism, invasion and metastasis [29-31]. Furthermore, even the CA9 and GLUT1, which play crucial roles in tumor cell metabolism associated with tumor survival, did not show survival significance in our research. Survival significance of CA9 expression has been studied in cervical cancer but this remains unclear [12,32,33]. In the terms of GLUT1, research on its survival outcome in cervical cancer remains preliminary with very few reported cases in other cancers [34].

There are numerous potential sources of the conflicting results of hypoxia markers and prognostic significance in cervical cancer. Foremost, sampling site is a factor to be considered. Hypoxia increases as the distance of the sampling site from blood vessel increase, and this contributes to conflicting results. HIF-1α is stabilized in the cytoplasm under hypoxic conditions and translocated to the nucleus as it dimerizes with HIF-1β binding to the hypoxia response element. Degree of cytoplasmic and nuclear expression often varies with different HIF-1α antibodies used which result in mixed and conflicting outcomes. Daponate et al. suggested applying a panel of HIF-1α antibodies to overcome controversy [35], however this approach introduces even more variables, and makes the application in a clinical setting unappealing. Medical condition of the patient can also affect the expression of hypoxia and metabolic markers. Patients with low level of hemoglobin and hyperglycemia in diabetes mellitus exhibited over-expression of HIF-1α and increased GLUT1 expression, respectively [36,37]. Manual scoring is time consuming, results in qualitative results of limited dynamic range, and is prone to intra- and inter-pathologist variability. The use of image analysis alleviates the issues of variability, and allows the application of quantitative scoring. The challenge with automated scoring is ensuring the correct cells are being quantitated, and many users rely on manual annotations of regions of interest. Computer-aided image analysis is limited by the same factors that manual interpretation are, mainly the quality of the immunohistochemical staining, and the overall quality of the tissue being stained [38].

Although HIF-1α expression was significantly higher in precancerous and cancer tissue than in normal tissue which was acquired around cancerous tissue, weak or moderate expression was observed in a large number of normal tissues. This finding was also observed in other study including IHC results of esophageal cancer [39]. Hypoxia may influence tissues around the cancerous lesions through increased interstitial pressure or metabolic product to affect oxygen release and consumption [39]. Similar to our result, Birner et al. reported HIF-1α was highly expressed in high grade CIN and cervical cancer compared to that in normal cervix but no difference was observed between high grade CIN and cancer [29]. Considering such high expression of HIF-1α from high grade CIN, HIF-1α is thought to be involved in early event of tumorigenesis. Since human papillomavirus (HPV) infection is involved in tumorigenesis, much research has been conducted in finding the association between HPV infection and HIF-1α. Lu et al. reported double transgenic mice with HPV 16 and HIF-1α showed highly invasive cervical cancer compared to that in single transgenic mice only with HPV 16 expression [40]. HIF-1α expression with HPV infection facilitates tumor progression from premalignancy to malignancy [40]. Furthermore in other study, HPV E6 oncoprotein, inactivator of p53 tumor suppressor gene, was shown to enhance HIF-1α stability and HIF-1-dependent vascular endothelial growth factor (VEGF) expression in hypoxia [41]. Considering our finding and together with previous studies, stabilization and expression of HIF-1α through high-risk HPV infection is speculated to play an important role in cervical cancer progression. In addition to HIF-1α, c-Met may co-facilitate cancer progression with HPV infection as well. In CIN and anal intraepithelial lesion (AIN), c-Met expression was correlated with oncogenic HPV infection but was not correlated with non-oncogenic HPV infection, condyloma acuminata [42,43]. However, further research is required to clarify the association between oncogenic HPV and c-Met expression in cervical cancer progression.

In this study, we evaluated protein expressions of HIF-1α and its related markers using automated digital image analysis. Manual interpretation of IHC is highly subjective and may produce conflicting results among studies. In such cases, automated digital image analysis for the interpretation of IHC could serve as an alternative method in presenting reproducible, objective and quantitative measurements. As a result of its ability to predict response to Trastuzumab, which specifically targets human epidermal growth factor receptor 2 (HER2), the assessment of HER2 expression has been incorporated in the diagnostic work-up of breast cancer. Hence, 32.7% of the U.S. laboratories reported to perform image analysis for quantitation of HER2 in a survey conducted in 2008 [44]. At present, fluorescence *in situ* hybridization (FISH) is commonly used to clarify equivocal expression of HER2 by manual interpretation before administration of Trastuzumab. However, automated image analysis of HER2 has been reported to show excellent concordance to FISH results, while reducing time required for interpretation and being more user-friendly compared to FISH [15] and therefore, it may substitute FISH in determination of equivocal case. Despite of powerful advantages mentioned above, digital image analysis has not been widely applied to diagnostic work-up outside breast cancer. As previously applied in breast cancer research, when used in gynecologic cancer studies, digital image analysis is expected to improve protein expression analysis from IHC.

## Conclusions

In summary, we have examined the primary players in the hypoxia signaling pathway, by immunohistochemistry combined with automated digital image analysis, but confirming their interactions, as well as defining which proteins are associated with outcome. Among the tested markers, HIF-1α and c-Met were involved in lymph node metastasis and tumor stage. Furthermore, our results confirmed the co-expression of HIF-1α and c-Met in cervical cancer. Both HIF-1α and c-Met demonstrated poor overall survival in the univariate analysis, however in the multivariate analysis, poor overall survival was only associated with c-Met expression. We demonstrate that c-Met correlates with HIF-1α and is prognostic factor in survival in cervical cancer. This data suggest that drugs that target c-Met may have therapeutic utility in the treatment of invasive cervical cancer.

## Abbreviations

CA9: carbonic anhydrase 9; HIF-1α: hypoxia inducible factor-1 alpha; GLUT1: glucose transporter type 1; CIN: cervical intraepithelial neoplasia; CIS: carcinoma *in situ*; VHL: von Hippel-Lindau tumor suppressor; PI3K: phosphatidylinositol 3-kinase; MAPK: mitogen-activated protein kinase; IHC: immunohistochemistry; LVSI: lymphovascular space invasion; LN: lymph node; ROC: receiver operating characteristic; SCC: squamous cell carcinoma; VEGF: vascular endothelial growth factor; AIN: anal intraepithelial lesion; HPV: human papillomavirus; FIGO: international federation of gynecology and obstetrics.

## Competing interests

The authors declare that there is no competing interests.

## Authors’ contributions

BWK, HC, J-HK and SMH conceived of the study and devised the experimental design. J-HK and SMH designed and build the tissuemicroarrays. BWK, HC, J-YC, and YK performed experiments. BWK, HC, J-YC, CC, J-HK and SMH performed data analysis for experiments or clinical records. BWK, HC and J-YC drafted the final version of the manuscript and figure legends. J-HK and SMH revised the figures, added critical content to the discussion and was responsible in revising all portions of the submitted portion of the manuscript. All authors read and approved the final manuscript.
